# Protein kinase C targeting of luminal (T-47D), luminal/HER2-positive (BT474), and triple negative (HCC1806) breast cancer cells in-vitro with AEB071 (Sotrastaurin) is efficient but mediated by subtype specific molecular effects

**DOI:** 10.1007/s00404-022-06434-2

**Published:** 2022-03-17

**Authors:** Veruschka Albert, Gerhard Piendl, Dali Yousseff, Hedwig Lammert, Michael Hummel, Olaf Ortmann, Wolfgang Jagla, Andreas Gaumann, Anja K. Wege, Gero Brockhoff

**Affiliations:** 1 Department of Gynecology and Obstetrics, University Medical Center Regensburg, Franz-Josef-Strauß-Allee 11, 93053 Regensburg, Germany; 2 Institute of Pathology, Kaufbeuren, Germany; 3 Institute of Pathology, Charité-Universitätsmedizin Berlin, Berlin, Germany

**Keywords:** Protein kinase C, AEB071, Sotrastaurin, Breast cancer

## Abstract

**Purpose:**

Protein kinase C (PKC) plays a pivotal role in malignant cell proliferation, apoptosis, invasiveness and migration. However, its exploitation as therapeutic target in breast cancer has been merely explored. Here were evaluated the AEB071 (Sotrastaurin™) treatment efficiency of breast cancer cell lines derived from estrogen receptor positive (T-47D), estrogen/HER2 receptor positive (BT474), and triple negative (HCC1806) breast cancer cells under 2D (monolayer) and 3D (multicellular tumor spheroids) culture conditions. Additionally, spheroid cocultures of BC and N1 fibroblasts were analyzed.

**Methods:**

We quantitatively assessed the proliferation capacity of breast cancer cells and fibroblasts as a function of AEB071 treatment using flow cytometry. The activities of PKC isoforms, substrates, and key molecules of the PKC signaling known to be involved in the regulation of tumor cell proliferation and cellular survival were additionally evaluated. Moreover, a multigene expression analysis (PanCancer Pathways assay) using the nanoString™ technology was applied.

**Results:**

All breast cancer cell lines subjected to this study were sensitive to AEB071 treatment, whereby cell proliferation in 2D culture was considerably (BT474) or moderately (HCC1806) retarded in G0/G1 or in G2/M phase (T-47D) of the cell cycle. Regardless of the breast cancer subtype the efficiency of AEB071 treatment was significantly lower in the presence of N1 fibroblast cells. Subtype specific driver molecules, namely IL19, c-myb, and NGFR were mostly affected by the AEB071 treatment.

**Conclusion:**

A combined targeting of PKC and a subtype specific driver molecule might complement specified breast cancer treatment.

## Background

Protein kinase C (PKC) activity has been implicated in the regulation of malignant cell proliferation, apoptosis, and tumor invasiveness [1]. It has been classically considered as a promoter of proliferation and aggressiveness of breast cancer (BC) [2]. However, PKCs have been described not per se to drive BC growth and progression. Instead, tumor suppressive PKC activity has been discovered as well. Moreover, presence of PKC and its activity in malignant cells and tissues has been found both increased [3, 4] and decreased [5, 6] compared to expression levels observed in normal (i.e., non-malignant) breast tissues. Understanding the importance of PKC expression and activity in BC is even more difficult in consideration of the expression pattern of a number of PKC isoforms. Three groups of PKCs, more specifically so called classical (PKCα, β1, β2 and γ), novel (PKCδ, ε, η, and θ), and atypical (PKCζ and ι) PKC isozymes are known to be differentially expressed in BC [1]. All of them show pleiotropic activity. Amongst all PKCs, the PKCδ isoform seems to play a special role probably both in normal and malignant tissues (incl. BC) because this isozyme is considered to mediate either growth stimulation or inhibition, which is dependent on the molecular and cellular context [7]. Thus, it is challenging to design isozyme-specific modulators (stimulators or inhibitors) that can be used as therapeutic agents.

Notwithstanding the rather complex and incompletely understood expression patterns and isozyme activities, evidence arose that above all the canonical PKCα has pro-survival, pro-proliferative, and pro-migration activity of BC cells in-vitro and independently predicts a poor 10 years outcome of BC disease [2]. This finding seems to be primarily valid for hormone (i.e., estrogen) receptor positive BCs. Accordingly, a number of PKC inhibitors (and siRNA based strategies) have been introduced into clinical trials for the treatment of human cancers [8, 9].

A not entirely new, but in the context of cancer not deeply and for the treatment of BC hardly ever explored, orally administered and putative potent inhibitor of several PKC isotypes is AEB071 (Sotrastaurin™, Novartis AG, Basel, Switzerland). It seems to have strong and specific activity on PKCα, PKCβ2, and PKCθ but a lesser impact on PKCδ, PKCε, and PKCη. In non-cancer related studies AEB071 has been shown to inhibit effector T-cell proliferation and function and therefore to have an immunosuppressive activity [10, 11]. Its usability to curb allograft rejection upon kidney transplantation has been positively evaluated [12–14]. However, the potential antitumor activity of AEB071 is less investigated. Only few studies revealed an anti-tumorigenic effect of AEB071 on e.g., different melanoma subtypes [15–18] and on cells derived from a variety of malignant hematological diseases [19–21]. One particular study is known by which PKCθ has been described to promote growth factor independent growth, anoikis resistance, and migration (in 3-D Matrigel and breast primary tumor xenografts) in a subset of triple negative breast cancer (TNBC) cells [22]. The same study revealed that cell treatment with AEB071 predominantly impairs the activity of PKCθ (rather than classic PKCs) and thereby reduces those tumor promoting effects. However, treatment efficacies in malignant cells derived from different taxonomic BC subtypes and related molecular mechanisms have not been explored by comparative analyses yet.

Here we analyzed the efficiency of AEB071 treatment in estrogen receptor (ER) positive (T-47D), ER/Human Epidermal Growth Factor Receptor 2 (HER2) double positive (BT474), and ER/HER2 negative (HCC1806) BC cell lines in 2D (i.e., monolayer) and 3D cell culture, the latter referred to as multicellular tumor **s**pheroids (MCTS). To this end we quantified the proliferation capacity of the cells exposed to 5 and 20 µM AEB071 by flow cytometry. A detailed quantification of cell cycle fractions (G0/G1-, S-, and G2/M phases) enabled the identification of the specific impairment of the cell cycle progression caused by AEB071 treatment. In order to simulate the in-vivo situation of tumor growth by the presence of stromal components somewhat better, we took advantage of heterologous 3D cocultures (COCUs) consisting of interacting tumor and fibroblast (N1) cells [23–25]. We quantified the impact of AEB071 treatment on PKC isoforms and intracellular key regulators of cell proliferation and survival/apoptosis by Western Blotting. Finally yet importantly, we applied the multiplex gene expression analysis nCounter PanCancer Pathway by nanoString™ and evaluated the regulation of 13 cancer-associated canonical pathways involved in malignant cell growth as a function of AEB071 treatment.

We found that AEB071 treatment is efficient in all cell lines tested in this study but the treatment efficiency is caused by affecting different subtype specific key molecules, above all IL19, c-myb, and NGFR, respectively.

## Methods

### Cell culture, treatment, and harvest

All cell lines used in this study were currently authenticated by the German Collection of Microorganisms and Cell Cultures GmbH (DSMZ, Braunschweig, Germany).

2D monolayer (ML) culture: ER/HER2 double positive BT474 (American Type Culture Collection no. HTB-20), ER-positive T-47D (ATCC no. HTB-133), and HER2/hormone receptor negative HCC1806 (ATCC no. CRL-2335), breast cancer cell lines as well as human fibroblast cells N1 (derived from the adult skin of a healthy donor [24, 26]) were used for treatment studies and pathway analyses. The cells were incubated with DMEM supplemented with 5% FBS (BT474, T-47D) or 10% FBS (N1). Only HCC1806 cells were seeded into RPMI supplemented with 5% FBS. In order to generate ML cultures 120.000 BT474, 80.000 T-47D, 40.000 HCC1806, and 80.000 N1 cells were seeded into 6 well plates using 2 ml medium. The used cell numbers were adapted due to the cell line specific doubling time and to avoid growth confluence during incubation time. Three days upon initial seeding the culture medium was refreshed and cells were treated with either 5 or 20 µM AEB071 (Sotrastaurin™, Novartis AG, Basel, Switzerland; stock solution solved in DMSO, Sigma-Aldrich, Taufkirchen, Germany, final conc. 0.01%) for 48 h. Initial titration experiments revealed half-maximal inhibition of cell proliferation by exposing BT474 cells to 5 µM AEB071. Maximum effects were seen when 20 µM AEB071 was used while the application of higher AEB071 concentrations did not result in more pronounced effects. Untreated or DMSO only (0.1 µM) treated cells were used as control. Cell numbers derived from ML cultures were counted upon harvesting and cell doubling times (t_g_) were calculated based on the following equation: t_g_ = (log 2 × t)/(log N – log N_0_), whereas “t” is the duration of cell incubation, “N” is the number of cells at end of incubation time, and “N_0_” is the number of cells seeded.

3D MCTS culture: 96 well plates were coated with agarose to prevent cell attachment. 2.000 BT474, T-47D, and HCC1806 breast cancer cells and 5.000 N1 fibroblasts were seeded in a volume of 200 µl per well. Because of the lower growth rate under 3D compared to 2D culture conditions the growth medium was supplemented with 10% FBS for all cell types. Cell treatments were done as described for 2D ML cultures. To avoid the development of necrosis in inner MCTS regions the spheroid growth was constrained by a limited number of cells seeded and a limited incubation time.

3D COCU: all cell lines were seeded into 96 well plates and initially incubated separately to enable the formation of MCTS and N1 based fibroblast spheroids [24, 27]. 4 days later N1 spheroids and MCTS were put together and incubated for 24 h which allowed cells to form interaction COCUs. Afterwards the mixed 3D cultures were treated as described above. Upon MCTS and COCU harvest the 3D cultures were pooled and exposed to trypsin and EDTA and thereby disaggregated.

All analyses were performed in triplicates.

### Flow cytometric proliferation assessment

Upon harvesting by trypsinization and separation the cells were washed twice with PBS fixed and permeabilized in cooled in MeOH (70%). After overnight incubation the alcohol was removed by washing twice with PBS. Afterwards the cells were incubated for 20 min in the presence of RNAase at 37°C and finally stained with 1 μg/ml DAPI 30 min prior to analysis. 1 × 10^5^ DAPI stained cells of every sample were analyzed using a BD FACSCanto-II™ Flow Cytometer (BD Biosciences, San Jose, USA) driven by the FACSDiva™ software v7.0 (BD Biosciences). Monocultures were DAPI stained only. In contrast mixed fibroblast/tumor cell samples derived from COCUs were additionally stained against the epithelial marker cytokeratin (pan anti cytokeratin, AF647 conjugated, BioLegend, San Diego, CA USA) which upon cell gating allowed to assess cell proliferation of tumor cells and fibroblasts separately.

DNA histograms were plotted on a linear scale and cell cycle fractions, i.e., percentages of cells in G0/G1-, S- and G2/M-phase, were quantified using the ModFit LT 3.2 software (Verity Software House, Topsham, ME, USA) upon discrimination of cell doublets, aggregates, and debris via pulse processing. Cell cycle fractions of treated cells are calculated as absolute values and as percentage of cell cycle fractions of untreated cells.

### Western blotting

BC cell lines were treated in-vitro with 20 µM AEB071 for 24 and 48 h. DMSO treated cells were used as control samples. For total protein analysis cells were lysed in cell-lysis buffer (Cell Signaling, Danvers, MA, USA) supplemented with Halt™ Protease and Phosphatase Inhibitor Cocktail (Thermo Fisher Scientific, Bremen, Germany). Cells and cellular fragments potentially detached from the culture flask as a result from treatment were not discarded. Protein concentration was calculated with Pierce BCA protein assay kit (Thermo Fisher Scientific). Depending on protein size twenty µg protein per lane were separated in 10 or 15% SDS-PAGE under reducing conditions (mercaptoethanol) and blotted onto polyvinylidene difluoride (PVDF) membranes. Membranes were blocked with 5% BSA or 5% low-fat milk and 1% Tween for 1 h and then incubated overnight at 4°C using following primary antibodies (all Cell Signaling; 1:1000, unless indicated otherwise): phospho-p90RSK (#9335), phospho-AKT (#9271), phospho-MAPK (#4370), phosphor-S6RP (#4858), phospho-PKCα/β2 (#9375), phospho-PKCδ (#9374), phospho-MARCKS (#2741), NGFR (p75NTR, #8238) c-myb (#12319), IL19 (ab198925, abcam plc, Cambridge, UK, 1:1000). Depending on the molecule size of the protein of interest anti-β-actin (#AF441; Sigma-Aldrich; 1:20,000) or Rab11 (#5589; Cell Signaling; 1:2000) were used as loading controls. As protein size standard, PageRuler plus prestained protein ladder (Thermo Fisher Scientific, Bremen, Germany) was used. After washing the membrane, incubation with HRP-linked secondary anti-rabbit (#7074; Cell Signaling; 1:2000) was incubated for 1 h at room temperature (all first antibodies were rabbit derived). Finally, the blots were visualized using the SuperSignal west pico PLUS chemiluminescent substrate (Thermo Fisher Scientific) and analyzed by ImageQuant LAS 4000 mini imager (GE Healthcare, Buckinghamshire, UK).

### RNA extraction from ML and MCTS and nanoString™ targeted pan-cancer pathways assay

RNA from each cell line grown in ML and as MCTS was purified using RNeasy Micro Kit (Qiagen, Hilden, Germany) according to the manufacturer’s protocol. Cells were lysed directly in the well plates (ML) or after collecting several MCTS (same cell line and treatment) with provided RLT-buffer. To ensure complete cell lysis, the lysate was homogenized by passing it five times through a sterile needle. RNase-free DNase (also provided with the RNeasy Micro Kit) was used to prevent potential contamination with genomic DNA before the concentrated RNA sample was diluted in up to 14 µl water. Afterwards, the purity (determined by the ratio of absorbance at 260/280 nm) and concentration of the samples was determined by nanodrop (Peqlab Biotechnologie GmbH, Erlangen, Germany).

50 ng total RNA from individual samples was used for the multi-gene expression analysis performed on the nCounter platform (prep Station and Digital Analyzer nanoString™ technology, Seattle, USA). Data were generated based on the PanCancer Pathway panel designed by and available from nanoString™. The panel addresses the expression of 770 genes, amongst them 606 Pathway genes, 124 driver genes and 40 housekeeping genes, assigned to 13 molecular pathways namely Notch, Wnt, Hedgehog, TGFβ, MAPK, STAT, PI3K, RAS, Chromatin Modification, Transcriptional Regulation, DNA Damage Control, cell cycle and apoptosis. The platform quantifies mRNA transcripts on a single molecule level using a multiplexed hybridization system and digital readouts of fluorescent barcoded probes that are hybridized to each transcription. Transcript counts were normalized against reference transcripts which are known to show the least variance with the geNorm algorithm. Normalized data were analyzed and visualized (expression heat maps, boxplots and volcano plots) using nanoString’s nSolver software (Ver 4.0) applying basic and advanced analysis based on the Pathifier algorithm [28]. Only significantly (*p* value ≤ 0.05) up- or downregulated gene expressions were considered by further evaluation and interpretation. Pathway deregulation scores were assessed for AEB071 treated samples compared to untreated controls (*n* = 3, respectively).

## Results

### AEB071 treatment differentially impairs cell proliferation of ER-positive, ER/HER2 double positive, and ER/PR/HER2 negative BC cells *in-vitro,* whereby the presence of fibroblasts reduces the treatment efficiency

#### AEB071 treatment retards proliferation of BT474, T-47D, and HCC1806 BC cells grown in 2D ML whereas the impact on the cell cycle differs; N1 fibroblast proliferation is not attenuated

**Fig. 1:**
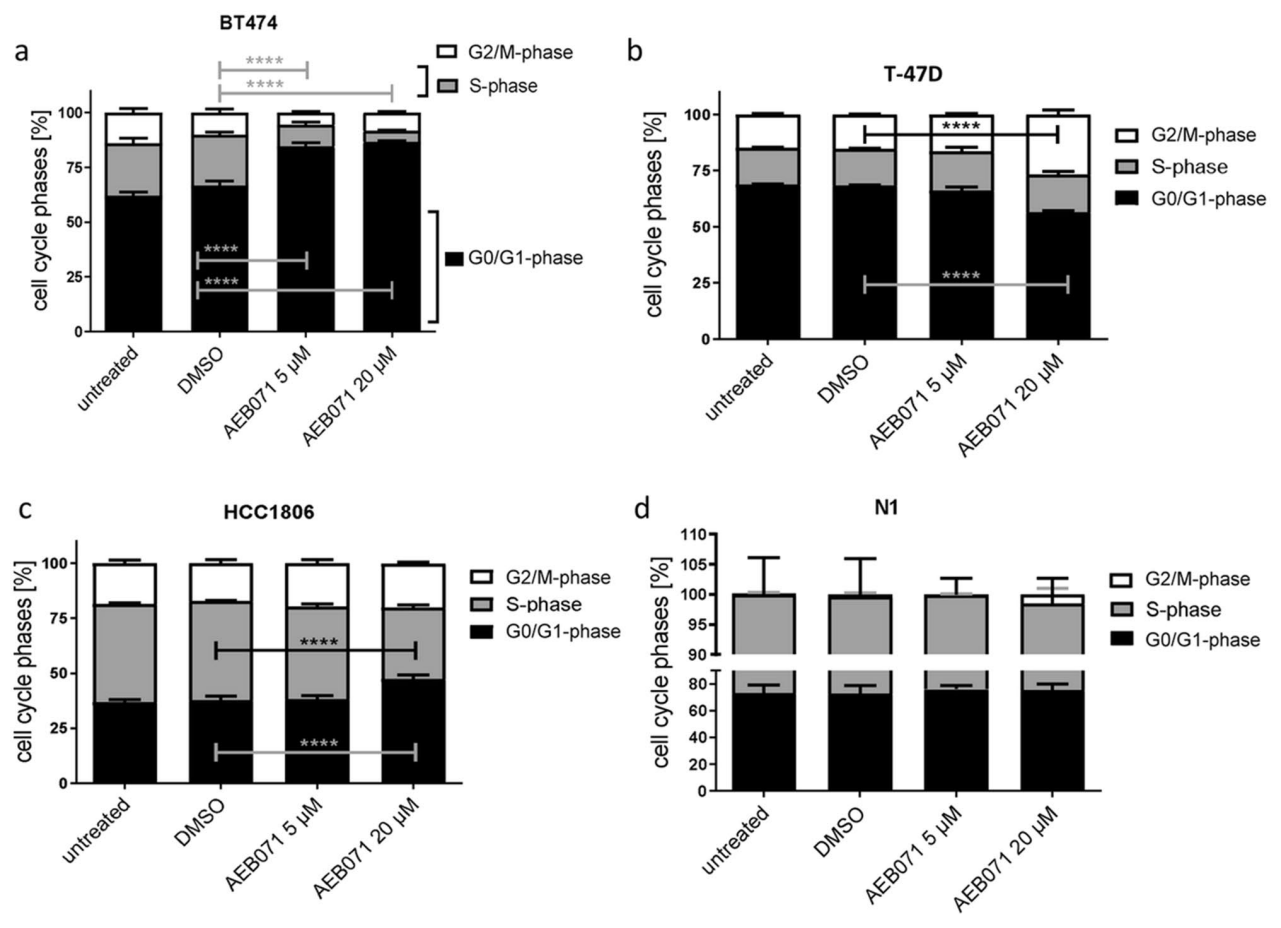
Distribution of cell cycle phases found in untreated or DMSO treated (both controls) and AEB071 (5 and 20 µM) treated BT474, T-47D, HCC1806, and N1 cells grown as ML. BT474 cells turned out as most sensitive to AEB071 treatment, which is reflected by a profound and highly significant decrease of the S-Phase fraction and a pronounced G1-phase compared to untreated cells. T-47D cells show an elevated G1-and G2-phase when exposed to 20 µM AEB071. Likewise, HCC1806 cells show only a response when exposed to 20 µM of the PKC inhibitor, reflected by as slight increase of the G1 cell fraction. N1 cells did not show any response to the treatment. Statistical analyses were done with the Dunnett's multiple comparisons test (DMSO as control; * = *p* ≤ 0.05; ** = *p* ≤ 0.01, ***p = ≤ 0.001). *n* = 3, data are shown as mean ± standard error of the mean (SEM)

In 2D ML culture BT474 cells turned out to be most sensitive to AEB071 treatment. When exposed to 5 and 20 µM the S-Phase fraction (SPF) was reduced from 24 to 10% and to even 5%, respectively (Fig. 1a). When exposed to 20 µM AEB071 the T-47D cell proliferation was retarded as well, which was evidently based on a considerable augmentation of the G2/M fraction (15% up to 27%) whereas the G0/G1 phase fraction was reduced (from 69 to 56%). Obviously, the exposition of T-47D cells causes a significant prolongation of the G2/M phase or even an arrest in G2/M (Fig. 1b). Untreated HCC1806 cells show the highest SPF with 45%. Accordingly, the G0/G1 phase fraction is relatively low with about 37%. HCC1806 cells did not respond to the application of 5 µM AEB071 and show only moderate sensitivity to 20 µM AEB071 treatment (Fig. 1c). The SPF of this cell line was reduced to 33% only in the presence of 20 µM of the PKC inhibitor, which goes along with an increased G0/G1 phase fraction (47%). In contrast to all BC cells the proliferation of N1 fibroblasts remained unaffected by AEB071 treatment; no significant alterations of cell cycle phases were seen (Fig. 1d). We substantiated the flow cytometric determination of cell cycle fractions by calculating cell doubling times of ML cultures as a function of treatment. Cell doubling times of target BC cells are given in Table 1. Basically, the most pronounced inhibition was seen when cells were exposed to 20 µM AEB071, while the cell doubling time was extended by 45.5% (BT474), 81.7% (T-47D), or 24.1% (HCC1806), respectively.

**Table 1:** Cell doubling times in days of BT474, T-47D, and HCC1806 BC target cells grown as ML as a function of AEB071 treatment. In addition, % changes compared to untreated cells are given

Cell line	No treatment (ctrl)	AEB071 (5 µM**)**		AEB071 (20 µM)	
	Days	Days	Change	Days	Change
BT474	3.93	4.27	+ 8.7%	5.72	+ 45.5%
T-47D	2.35	2.5	+ 6.4%	4.27	+ 81.7%
HCC1806	1.41	1.46	+ 3.55%	1.75	+ 24.1%

### AEB071 treatment retards proliferation of BT474 and T-47D BC cells grown 3D MCTS, whereas HCC1806 cells are almost insensitive in 3D culture

**Fig. 2:**
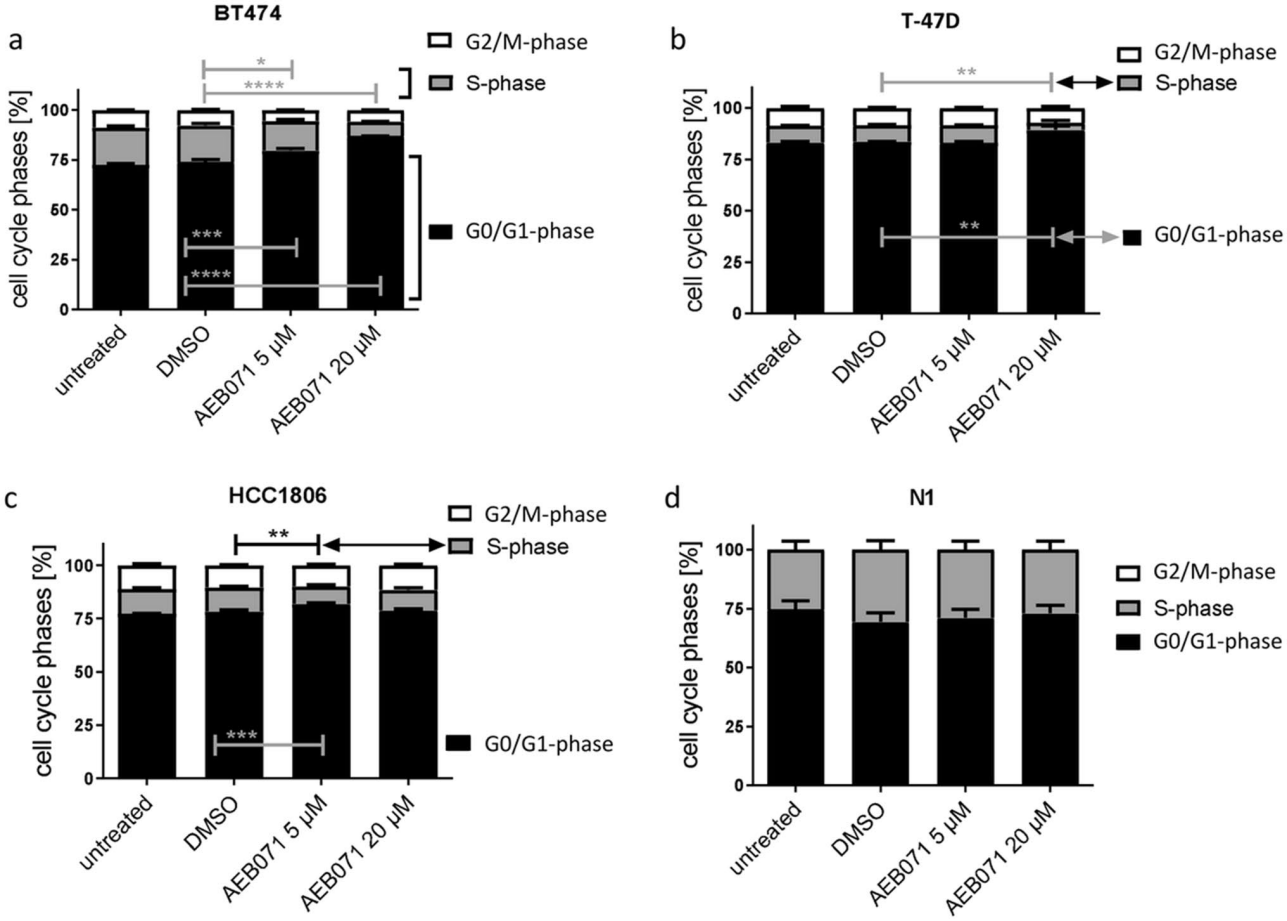
Distribution of cell cycle phases found in untreated or DMSO treated (both controls) and AEB071 (5 and 20 µM) treated BT474, T-47D, HCC1806, and N1 cells grown 3D spheroids. Overall, the proliferation capacity of the BC cells was lower in 3D MCTS culture compared to 2D ML culture. Nevertheless, BT474 cells remained the most sensitive cells when exposed to AEB071. Compared to this finding, the AEB071 treatment of T-47D and HCC1806 cells resulted in only little inhibitory effects, although with smaller statistical significance. Again, N1 cells were insensitive. Statistical analyses were done with the Dunnett’s multiple comparisons test (DMSO as control; * = *p* ≤ 0.05; ** = *p* ≤ 0.01, *** = *p* ≤ 0.001).* n* = 3, data are shown as mean ± standard error of the mean (SEM)

The proliferation capacity of all three BC cell types in spheroids is inherently lower than in ML culture which is known to be due to a pronounced 3D related cell contact and a gradient of oxygen and metabolites (e.g., lactic acid and carbon dioxide) as a typical feature of MCTS [27]. Accordingly, the effectiveness of AEB071 treatment is basically smaller than in ML culture. Nevertheless, BT474 cells remain most sensitive to 20 µM AEB071 treatment when cultured as 3D MCTS and the SPF is reduced to 7% compared to 19% in untreated cells (Fig. 2a). The SPF of T-47D cells was reduced from 8 to 4% by 20 µM AEB071 treatment (Fig. 2b) and the G0/G1 phase in T-47D cells increased upon AEB071 treatment, which was not seen in 2D monolayer cells. The most striking effect of untreated HCC1806 cells in 3D culture is the considerably reduced SPF compared to ML culture (12% vs. 45%). Moreover, HCC1806 cells do not show a considerable response to AEB071 treatment when incubated as 3D MCTS. Only a little effect was seen upon 5 µM AEB071 treatment (SPF is reduced from 12 to 8%) which was, however, nearly not seen upon the exposition to 20 µM AEB071 (SPF 10%). Overall, HCC1806 cells were almost completely insensitive when incubated as MCTS (Fig. 2c). As already observed in 2D ML culture there was no effect of AEB071 treatment on N1 cells grown in 3D spheroids (Fig. 2d).

#### The presence of N1 fibroblasts in 3D culture further attenuates the sensitivity of BC cells to AEB071 treatment

**Fig. 3:**
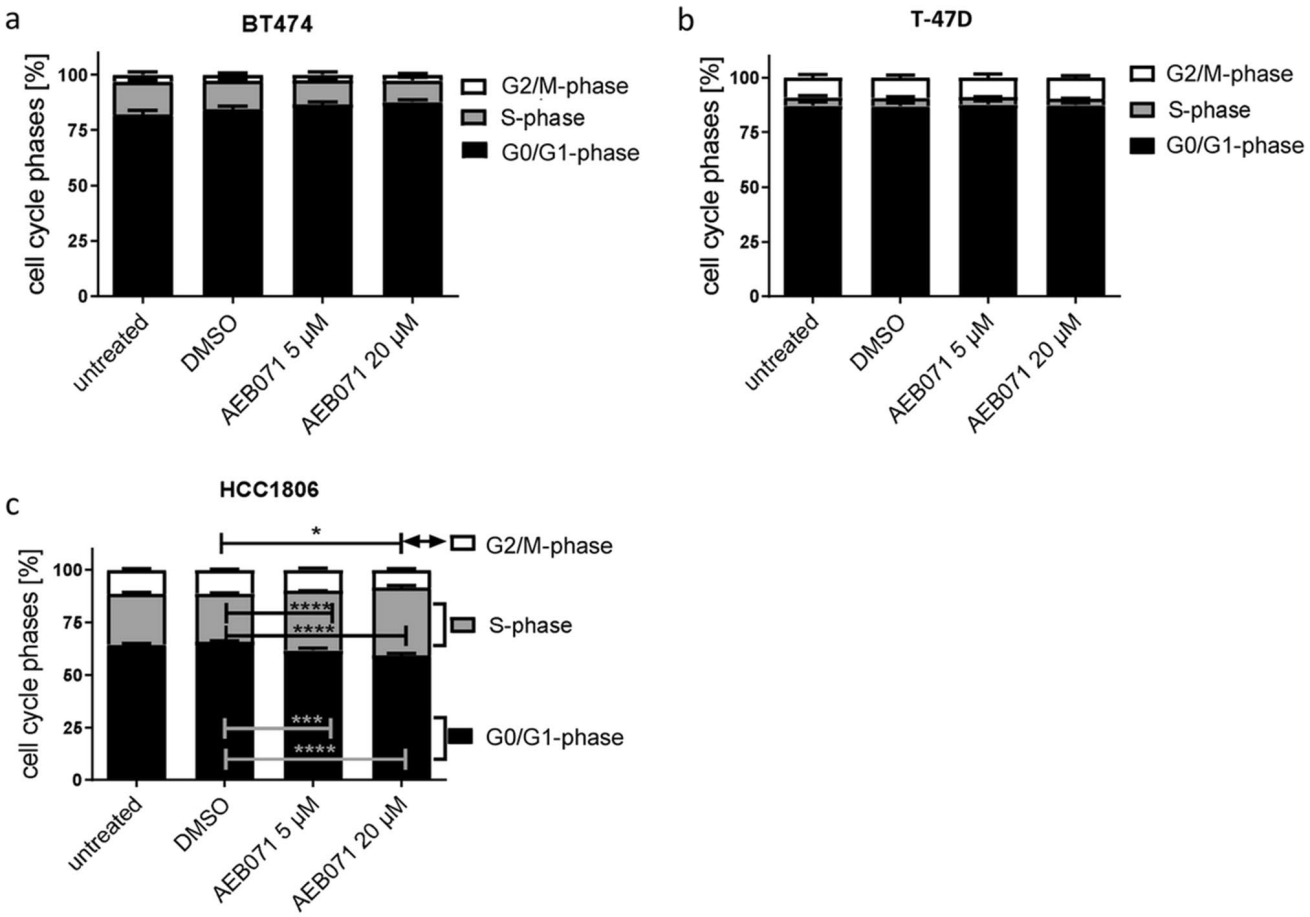
Distribution of cell cycle phases found in untreated or DMSO treated (both controls) and AEB071 (5 and 20 µM) treated BT474, T-47D, HCC1806, and N1 cells grown as COCU, i.e., in the presence of N1 fibroblasts. When incubated in the presence of N1 fibroblasts the sensitivity of ER-positive BT474 and T-47D cells to AEB071 treatment was completely abolished. Only in HCC1806 cells a significant, but very little effect of AEB071 treatment could be seen. Statistical analyses were done with the Dunnett’s multiple comparisons test (DMSO as control; * = *p* ≤ 0.05; ** = *p* ≤ 0.01, *** = *p* ≤ 0.001). *n* = 3, data are shown as mean ± standard error of the mean (SEM)

In general, the proliferation capacity of BC cells under 3D conditions is further reduced in coculture with N1 fibroblasts (Fig. 3). Consequently, the AEB071 treatment effect in 3D coculture is not as pronounced as in 2D culture (Fig. 1), nor as in 3D MCTS (Fig. 2). More specifically, the SPF of BT474 MCTS cells exposed to 20 µM is indeed diminished from 15 to 10% in the presence of N1 cells, however, without statistical significance (Fig. 3a). T-47D do nearly not proliferate in 3D coculture (SPF about 4%) and accordingly do nearly not respond to AEB071 treatment (i.e., no measurable treatment effect, Fig. 3b). Thus, the desensitizing effect of N1 cells is most pronounced in luminal T-47D cells. Interestingly, in HCC1806 cells the SPF fraction is apparently higher in COCU (28% and 32% when treated with 5 and 20 µM, respectively) than in untreated MCTS (24%). Nevertheless, the inhibitory effect of AEB071 on HCC1806 in 3D coculture (i.e., in the presence of fibroblasts) is similar small to the effect seen in HCC1806 cells grown as MCTS. (Fig. 3c).

#### AEB071 treatment of BT474, T-47D, and HCC1806 cells causes a nearly identical distribution of differentially expressed genes, however the most highly downregulated genes differ and are apparently subtype specific

**Fig. 4:**
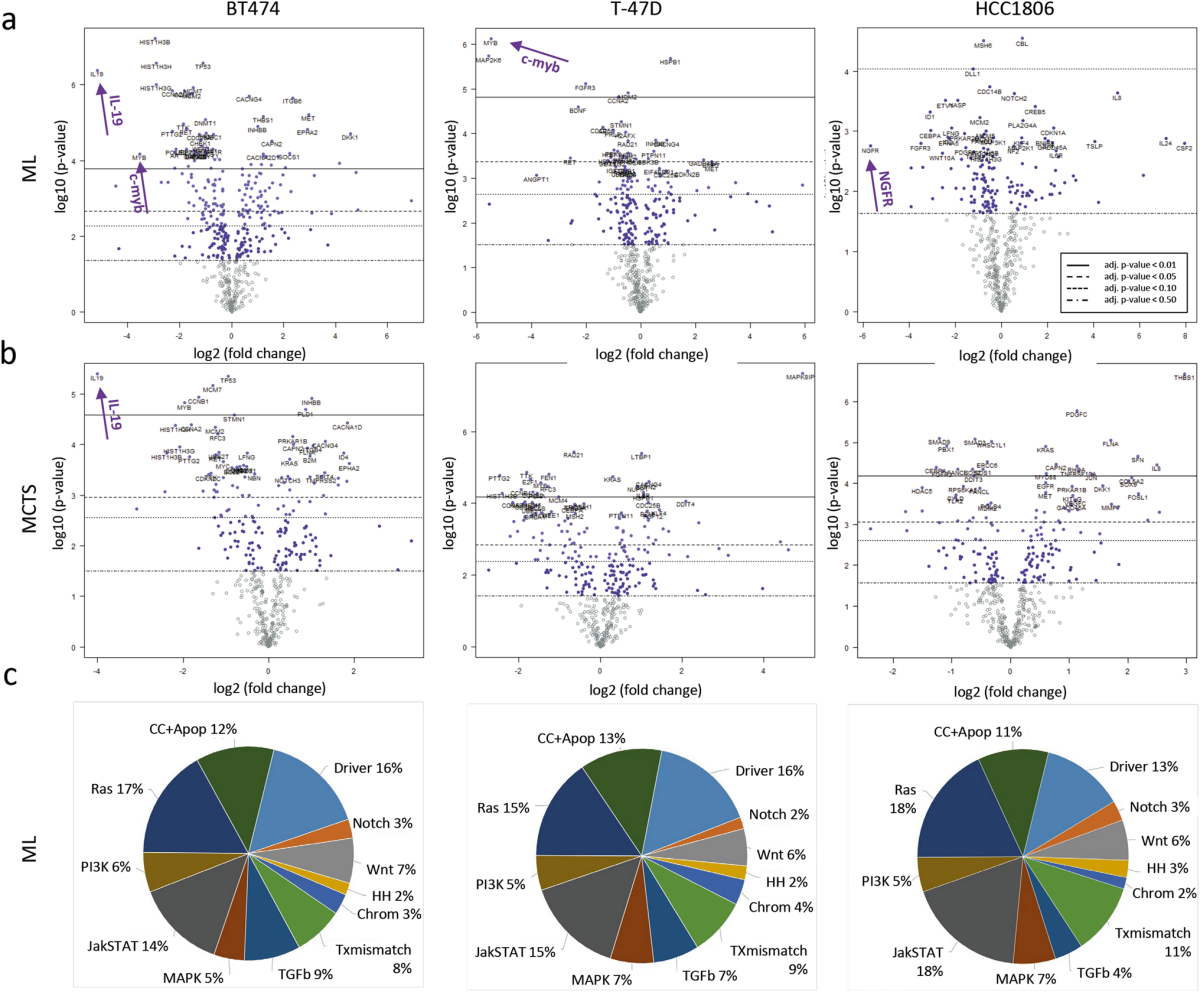
**A** and **B** Volcano plots showing differential gene expression of 730 mRNA analytes of the nCounter PanCancer nanoString assay as a function of ML and MCTS treatments with AEB071 (*n* = 3). DMSO treated cells served as reference. Data are displayed as log2 fold changes (x-axes) vs. significances of changes (y-axes) expressed as log10 p-values. Different significance levels (*p* ≤ 0.01, 0.05, 0.10, 0.50) are indicated by solid, dashed, and dotted lines (see insert in the right plot of panel A) as well as purple colored dots. Genes most heavily downregulated with pronounced significance are marked by an arrow (i.e., BT474 ML: IL19, c-myb; BT474 MCTS: IL19; T-47D: c-myb, HCC1806: NGFR). **C** Distribution of differentially expressed genes in BT474, T-47D, and HCC1806 ML cells caused by AEB071 treatment. Gene expressions were quantified using the PanCancer gene panel (*n* = 730) compiled and designated to different molecular pathways by nanoString Technologies. Only significant changes (*p* ≤ 0.05) of gene expressions were included into the pie charts: Number of significantly affected genes were in BT474: *n* = 275, in T-47D: 209, in HCC1806: 161

Multiplexed gene expression analysis was performed in response to AEB071 treatment of ML and MCTS cultures. Advanced nSolver based principal component and gene set analyses (visualized by expression heat maps) revealed a good consistency of not treated and AEB071 treated samples (*n* = 3, respectively). Gene expression patterns clustered considerably with respect to treatment modality (AEB071 vs. control, data not shown). Here, absolute changes of gene expressions are displayed in volcano plots (Fig. 4a and b) illustrating that a variety of gene expressions are up- und downregulated, whereas the smaller fraction of genes achieved significant changes. The nSolver based gene expression analyses revealed that regarding the total PanCancer panel (*n* = 730) 275 gene expressions in BT474, 209 expressions in T-47D, and 161 expressions in HCC1806 were significantly (i.e., *p* ≤ 0.05) modified upon treatment of ML cells with AEB071 (Fig. 4c). The most severely downregulated expressions were IL19 in BT474, c-myb in BT474 and T-47D, and NGFR in HCC1806 cells. Nevertheless, a major share (i.e., about one third) of affected genes plays an essential role in the regulation of cell proliferation and survival or are known as drivers of malignancy (Fig. 4c). About another one-third (30–36%) of affected genes are verifiably involved in the JAK/STAT and/or Ras signaling. Considering the 730 genes attributed to twelve sets as part of the PanCancer assay it is apparent that the overall pattern of affected gene sets does not differ but is nearly identical amongst the three cell lines used in this study.

#### IL19, c-myb, and NGFR genes are the most affected genes upon AEB071 treatment

**Fig. 5:**
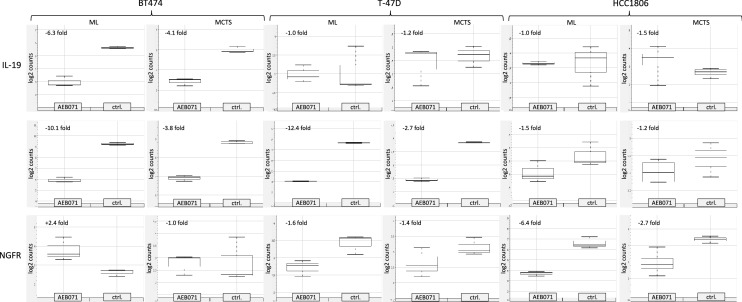
Box plots of most pronounced downregulated gene expressions in BT474, T-47D, and HCC1806 BC cells incubated as ML and MCTS and exposed to the PKC inhibitor AEB071. Individual gene regulations were identified using the nSolver Analysis Software (nanoString Technologies). The degree of downregulation is given as numerical values (log twofold changes) in the respective plots. nSolver gene expression analysis revealed IL19 in BT474, c-myb in T-47D, and NGFR in HCC1806 as most pronounced downregulated genes

The absolute fold change of genes that show the most pronounced downregulation with highest significance were calculated separately for individual BC cell lines. The results are displayed as box plots in Fig. 5. Accordingly, we found in ML culture a 6.3-fold downregulation of IL19 in BT474 cells, a 12.4-fold downregulation in of c-myb in T-47D cells, and a 6.4-fold downregulation of NGFR in HCC1806 cells. The downregulation of these genes was considerably less affected in the other BC cell lines, respectively.

### Alteration of intracellular signaling pathways as a function of AEB071 treatment

#### The activity of PKCα, β, and δ isoforms as primary AEB071 targets and MARCKS as primary PKC substrate were remarkably reduced upon AEB071 treatment

**Fig. 6:**
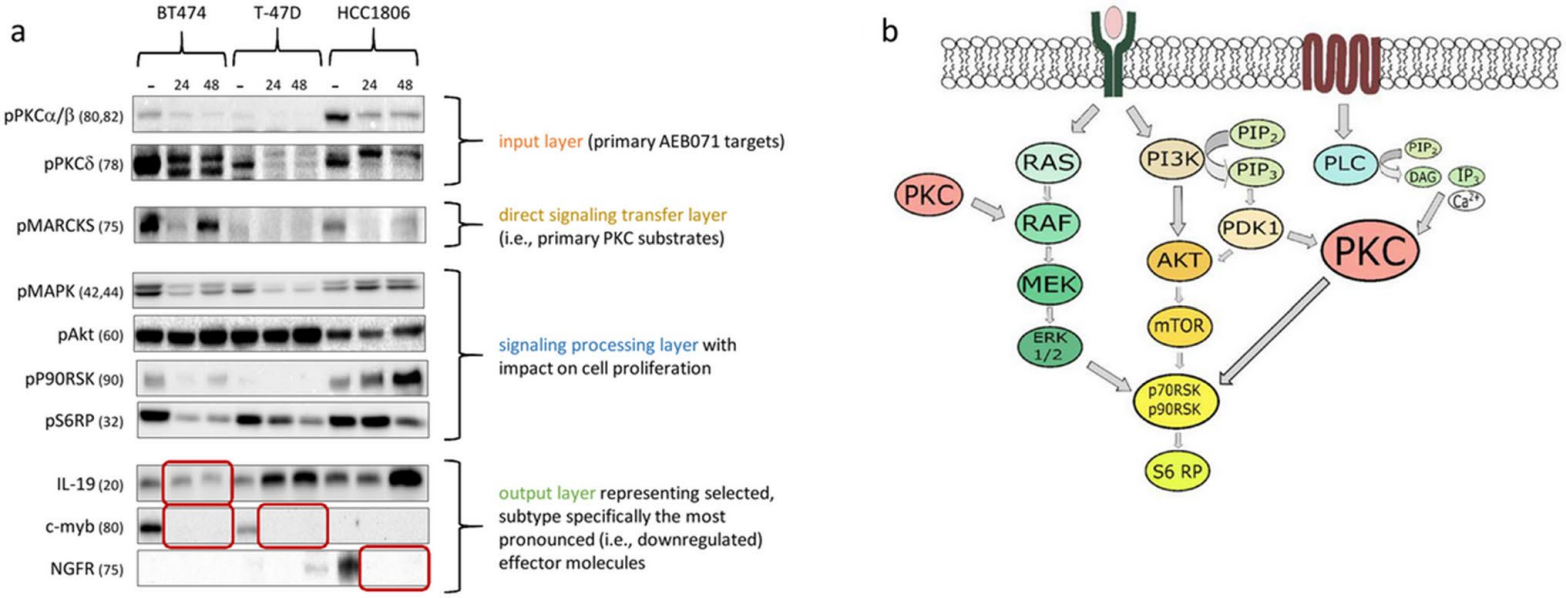
**A** Western Blots of key target and signaling molecules affected by AEB071 treatment. The annotation “-” indicates untreated control samples, and “24” and “48” the duration of treatments in hours. Numbers indicate molecular sizes in kDa. Although the total levels of phosphorylated PKCα/β and PKCδ differ in the three untreated cell lines, the phosphorylation of these PKC isotypes is decreased upon AEB071 treatment independently of the subtype. Accordingly the activity of primary PKC substrates i.e., MARCKS molecules is decreased as well, only in BT474 cells MARCKS molecules seem to become reactivated after 48 h. Further downstream the phosphorylation of MAPK is remarkably reduced upon AEB071 treatment in BT474 and T-47D cells but not in HCC1806 cells. The same is valid further downstream for P90RSK in BT474 and T-47D cells but not in HCC1806 cells. The activity of Akt is not affected by AEB071 treatment in all cell types. The most downregulated effector molecules (as identified by the nanoString technology) were investigated on the protein level and a corresponding protein downregulation could be verified for IL19 in BT474, c-myb in BT474 and T-47D, and NGFR in HCC1806 cells (as indicated by red rectangles). **B** A proposed model of intracellular signaling that causes attenuated cell proliferation upon AEB071 treatment. The ERK1/2 and the PI3K/Akt pathways are known to predominantly drive cell proliferation in many cell types, also in breast cancer cells. While the activity of key molecules of the ERK1/2 pathway (i.e., MAPK itself) but not of the PI3K pathway (i.e., Akt) causes an inactivation of the common downstream molecules P90RSK and S6RP, it can be assumed that the PKC targeting mainly affects the MAPK pathway in BT474 and T-47D but not the PI3K/Akt pathway. In contrast, HCC1806 cells that are barely AEB071-sensitive do not show any weakened activity of the MAPK or the PI3K pathway. Nevertheless, the most affected effector molecules (i.e., IL19, c-myb, NGFR) differ in all three cell types investigated in this study

We analyzed the activation state of three essential PKCs (i.e., α, β, and δ) in ML culture as primary targets of the AEB071 inhibitor (Fig. 6a). Due to the missing availability of pPKCα and β specific detection antibodies the phosphorylation of PKCα and β was detected simultaneously by a single detection antibody. As expected we found a reduced phosphorylation of PKCα/β in all three cell lines which was visible after 24 h and 48 h of AEB071 treatment, respectively. In all BC cell lines used in this study the PKCδ is apparently expressed at pronounced levels and AEB071 treatment causes a detectable reduction of PKCδ phosphorylation in all cell lines subjected to this study (Fig. 6a). Though this effect was mostly pronounced in T-47D cells since the phosphorylation of PKCδ vanished nearly completely upon cell treatment.

MARCKS protein as primary PKC substrate represents the direct signaling transfer layer between the PKC targets and further downstream signaling molecules [29]. Here we found the strongest activity of MARCKS in BT474 cells in the absence of AEB071 (Fig. 6a). Nevertheless, the phosphorylation of MARCKS was reduced in all cell types by AEB071 treatment even though this was visible in BT474 cells particularly after 24 h. Within 48 h, the phosphorylation obviously reappears in this cell line.

#### AEB071 treatment attenuates predominantly the MAPK but not the PI3K pathway in ER-positive BC cell lines

As key molecules of two most important pathways driving the cell proliferation and vitality of epithelial (cancer) cells we evaluated the activities (i.e., the phosphorylation) of MAPK, Akt, P90RSK, and S6RP as representatives of the so called MAPK and the PI3K pathway. The MAPK activity is remarkably reduced in the two ER-positive cell lines BT474 and T-47D but not in HCC1806 cells (Fig. 6a). In contrast, in all three cell lines the phosphorylation state of Akt is not affected at all by the AEB071 treatment. Further downstream the phosphorylation of P90RSK is evidently reduced in BT474 and T-47D but definitely not in HCC1806. Instead, in this triple negative line a slightly enhanced pMAPK and a pronounced enhanced pP90RSK upon AEB071 treatment can be observed. The S6RP phosphorylation is first and foremost reduced in the ER-positive cell types and is less reduced in HCC1806 cells which is only seen after 48 h of AEB071 treatment. Overall, an inhibiting effect of AEB071 treatment on intracellular key molecules involved in “proliferation pathways” is particularly apparent in ER-positive cells (BT474, T-47D) and less pronounced in triple negative HCC1806 cells.

#### The most strongest downregulated biomarkers upon AEB071 treatment seem to be subtype specific

As identified by the nanoString technique the most strongly downregulated gene transcripts were IL19 in BT474, c-myb in BT474 and T-47D, and NGFR in HCC1806 cells. We performed Western Blotting to evaluate the respective protein expression accordingly (Fig. 6a). Indeed, we found IL19 protein downregulated only in BT474 cells while IL19 in T-47D and in HCC1806 cells rather tends to become even upregulated upon AEB071 treatment. Likewise, a strikingly reduced c-myb protein expression correlates with the significantly reduced quantity of corresponding RNA transcripts in BT474 and T-47D cells. c-myb expression was detectable neither in untreated nor in treated HCC1806 cells. Finally, the NGFR could only be visualized in untreated HCC1806 cells at a pronounced level. Upon AEB071 treatment the amount of NGFR was reduced to an undetectable level in these cells.

## Discussion

Aim of this study was to assess the efficiency of AEB071-based PKC targeting in ER-positive (T-47D), ER/HER2 double positive (BT474), and triple negative (HCC1806) BC cell lines subjected to 2D, 3D-mono, and 3D-coculture conditions. In order to evaluate the impact of a stromal environment on the treatment efficiency, tumor cells were incubated in the presence of human fibroblasts (N1) under 3D coculture conditions.

When exposed to AEB071 the proliferation of all BC cell lines used in this study was significantly attenuated, especially in 2D ML culture, whereas the application of 20 µM was generally more effective than the cell exposition to 5 µM AEB071. The attenuated cell proliferation of BT474 and HCC1806 cells grown as 2D ML is mainly due to a considerably reduced S-phase and an enhanced G0/G1-fraction while in T-47D cells a pronounced G2/M-phase has been observed. Overall, BT474 cells proved to be most sensitive and were responsive when exposed to 20 µM and even to 5 µM AEB071. A significant growth inhibition of HCC1806 cells was only seen when exposed to 20 µM AEB071 while the overall proliferation capacity, represented by a relatively high SPF, remained relatively high. Nevertheless, as shown by flow cytometric cell cycle analyses the inhibitory effect of AEB071 treatment results in a substantial reduction of cell multiplication of all BC cell lines analyzed in this study. This becomes especially evident with respect to the cell doubling times, that are significantly prolonged when BC cells were exposed to 20 µM AEB071.

A well-known phenomenon is the reduced proliferation capacity of tumor cells in 3D MCTS compared to ML culture. This can be attributed to a modified cell metabolism and microenvironment in inner spheroid compartments and is moreover due to a pronounced cell-cell contact [27]. Thus, cells grown in spheroids show a decelerated cell expansion compared to cells in 2D culture even without any treatment. Consequently, the AEB071 treatment effect expressed in absolute values is not as pronounced as under 2D conditions. Although the concentrations of 5 and 20 µM AEB071 used in this study are relatively high both concentrations are within the typical range of concentrations which have been applied for preclinical cancer treatment studies elsewhere [22, 30]. Nevertheless, an impeded penetration of AEB071 into inner MCTS compartments must be considered that results in a diminished accessibility of target cells [31]. Further evaluations addressing the tissue accessibility for AEB071 at a broader dose range could reveal appropriate concentrations to be applied in prospective preclinical in-vivo studies. Conspicuously, the sensitivity of the ER-positive BC cells, with (BT474) and without (T-47D) HER2 over-expression to the AEB071 treatment is considerably reduced in the presence of N1 fibroblasts. By contrast, the slightly enhanced S-phase fractions of HCC1806 cells in COCU compared to ML cells are somewhat ambiguous. Nevertheless, an attenuated HCC1806 proliferation when exposed to AEB071 has not been seen in COCU as well. Whether the impact of mesenchymal cells on ER-positive tumor cells in 3D culture is directly mediated by fibroblast-tumor cell interaction or by soluble components released by the N1 cells is unclear at the current stage of investigation. If the desensitizing effect is somehow linked to an ER pathway related signaling remains to be explored. Interestingly, N1 fibroblasts, when incubated in the absence of tumor cells, were basically insensitive to AEB071 treatment both under 2D and 3D culture conditions.

Western Blotting revealed that the application of AEB071 affects the total phosphorylation state of three PKC subtypes, namely PKCα, PKCβ2, and PKCδ, although the initial level of PKC phosphorylation (i.e., without AEB071 treatment) differed in the lines compared in this study. The most pronounced inhibition of PKCδ could be seen in T-47D cells. These data indicate the relatively unspecific targeting of PKCs, at least with regard to PKCα, PKCβ2, and PKCδ. In addition, the data suggest that the absolute expression level and phosphorylation state of PKCs is not indicative for a response to AEB071 treatment. The strongly reduced phosphorylation of the primary PKC downstream molecule MARCKS upon AEB071 treatment reflects that AEB071 inhibitory effect is independent of the cell type and obviously independent of the amount of the PKC isoform expression levels. Thus, the MARCKS protein seems to play a pivotal role in mediating a pro-proliferative effect in BC cells [29], which is considerably reduced by AEB071 treatment.

The analysis of selected intracellular pathway molecules known to be essential for the cell cycle progress revealed a combined reduction of MAPK, 90RSK, and S6RP phosphorylation in the luminal (i.e., ER-positive) BT474 and T-47D cells, but not in triple negative HCC1806 cells. This is compatible on the one hand with the high treatment sensitivity of BT474 and T-47D cells and on the other hand with the low sensitivity of HCC1806 cells. Since an active MAPK pathway is considered to drive the cell cycle transition at the G1/S-phase [32], downregulation of this signaling route contributes to the impaired cell cycle progress by a cell accumulation at late G1, while the T-47D cell cycle progress is additionally impaired in G2/M. The latter phenomenon has been previously seen in epithelial cells as well [33]. More specifically, the block of cell cycle progress of T-47D cells upon AEB071 treatment in G2/M rather than in G1/S can be attributed to a total shut down of PKCδ activity which is not seen in BT474 or HCC1806 cells when exposed to AEB071. One can conclude that PKCα preferably promotes the G1/S cell cycle transition, while PKCδ is predominantly engaged in the G2/M-G1 transition, which is compatible with previous reports [34].

The model in Fig. 6b illustrates a simplified scheme of the main pathways transferring pro-proliferative and pro-survival signaling. The model can on the one hand explain that an inhibited MAPK axis results in an inhibition of the downstream P90RSK molecule. This mechanism seems to take place in BT474 and T-47D cells. On the other hand, when neither the MAPK pathway nor the PI3K/Akt pathway gets impaired by the AEB071 treatment, the activity of P90RSK remains unaffected. This phenomenon seems to be valid in HCC1806 cells which would explain that this cell type is largely insensitive when exposed to AEB071. Notably, only the long-term (48 h) exposition of HCC1806 cells to 20 µM AEB071 caused a slight decrease of phosphorylated S6RP (which acts further downstream of P90RSK). This finding is in agreement with the only but limited inhibitory effect of the inhibitor at higher concentration (s. Figure 1c). Taken together, one might conclude that only an efficient inhibition of the key signaling molecule P90RSK, which is potentially triggered by both, the MAPK and the PI3K/Akt pathway, results in a significant retardation of cell proliferation.

Even though the impact of PKC isoforms on cell cycle regulation is undisputable [34], extended studies should not only address the proliferation capacity of target cells as a function of AEB071 treatment but should also include additional read-out parameters as for example cell vitality, cell damages and death, or even perhaps cell immunogenicity. Likewise, broadening of the present study to additional test models that represent specific BC subtypes (cell lines and in-vivo models) are required to further substantiate the findings of this study.

Multiplexed gene expression analyses by the nanoString™ technology revealed that a variety of signaling pathways are perturbed by AEB071 treatment but in particular those involved in the regulation of cell proliferation and propagation, as well as viability and apoptotic cell death. Basically, the pattern of affected pathway sets as designated by nanoString™ resembles amongst the three subtype specific cell lines when treated with the PKC inhibitor AEB071. However, the most severely molecules affected in BT474, T-47D, and HCC1806 cells are not identical and seem to play a key role in the respective taxonomic subtypes.

IL19, for example, is considerably downregulated in ER/HER2-positive BT474 cells only. In contrast, it is rather upregulated in T-47D and HCC1806 cells. It has been previously demonstrated that IL19 treatment stimulates cell proliferation and migration especially of ER-positive (MCF-7) BC cells in-vitro. Moreover, IL19 enhances tumor growth and metastasis of ER-positive BC in preclinical in-vivo models [35]. In humans, IL19 drives pathogenesis of BC and promotes tumor progression by paracrine and autocrine activities [35, 36]. Overall, there is evidence that IL19, either derived from the environment or by an autocrine release by tumor cells, seems to impair the course and clinical outcome of ER-positive BC disease.

The c-myb transcription factor is another marker that has been shown to play a pivotal role predominantly in ER-positive BC. Here we found that upon AEB071 treatment c-myb is considerably downregulated in hormone receptor positive BT474 and T-47D, but again not in ER-negative HCC1806 BC cells. A pronounced c-myb expression has been demonstrated in virtually all ER-positive tumors, whereas in other taxonomic subtypes c-myb is just rarely expressed or even undetectable [37]. Notably, a reduced c-myb expression has been shown to inhibit tumor cell proliferation of ER-positive but not ER-negative BC cells [38]. Thus, the transcription activity of c-myb seems to be essential particularly in luminal (i.e., ER-positive) BC. Since resistance to anti-estrogens such as tamoxifen is a significant problem in ER-positive BC a specific targeting of ER effectors like c-myb may be beneficial for the treatment of luminal BC. A combined application of anti-estrogens and anti-c-myb therapies might be a useful strategy to be tested in preclinical in-vitro and in-vivo models.

NGFR is most conspicuously downregulated as a consequence of AEB071 treatment in triple negative HCC1806, but not in hormone receptor positive BT474 and T-47D cells. Interestingly, triple negative and basal like BCs have been shown not only to express higher NGFR levels than other BC subtypes but the NGFR expression was also associated with a poor prognosis of BC disease [39]. NGF binding to BC cells via the NGFR has been shown to have mitogenic and anti-apoptotic activity [40] and a functional NGF/NGFR system apparently contributes to chemotherapeutic resistance in TNBC cells [41]. Thus, an AEB071 mediated downregulation of NGFR seems to impair a molecular key component that drives the growth of hormone receptor and HER2 receptor negative BCs. However, the usefulness of NGFR targeting in triple negative BC cells remains to be explored.

Finally, it is important to note, that the aforementioned most conspicuously affected molecules become coincidentally downregulated both on the transcriptional (mRNA) and the translational (protein) level. This finding stresses the respective importance of these molecules in subtype specific cell types subjected to this study, even though more than the most affected molecules and pathways as highlighted here might play a role for cell type specific sensitivity to AEB071 treatment.

## Conclusion

Overall, the simultaneous slow-down of various pathways seems to be associated with and even essential for a pronounced sensitivity to AEB071 treatment, while in particular those pathways regulating the cell cycle progress and apoptotic cell death are predominantly affected. More specifically, the highest sensitivity of BC cells to AEB071 treatment was seen when both the MAPK and the PI3K/Akt pathways are simultaneously disrupted. Although the primary molecular targets of AEB071 (i.e., PKC isoforms) are identical in all BC cells putative subtype specific driver molecules involved in tumor growth become massively downregulated upon AEB071 treatment. Thus, a tumor targeting against PKC and those subtype specific key molecules is potentially useful to complement the existing portfolio for individualized BC therapies. Nevertheless, further preclinical in-vitro studies and those based on appropriate in-vivo models are required to evaluate the suitability of PKC targeting in humans.

## Abbreviations

BC: Breast cancer
COCU: Coculture
ER: Estrogen receptor
HER2: Human Epidermal Growth Factor Receptor 2
kDa: kiloDalton
MCTS: Multicellular tumor spheroids
ML: Monolayer
NGFR: Nerve growth factor receptor
PVDF: Polyvinylidene difluoride
PKC: Protein kinase C
SEM: Standard error of the mean
SPF: S-phase fraction
TNBC: Triple negative breast cancer
